# Supplementary figures and images for: Traditional Thai Massage Promoted Immunity in the Elderly via Attenuation of Senescent CD4+ T Cell Subsets: A Randomized Crossover Study
Source: Int J Environ Res Public Health. 2021 Mar 19;18(6):3210. doi: 10.3390/ijerph18063210 (PMC8003732; doi:10.3390/ijerph18063210)

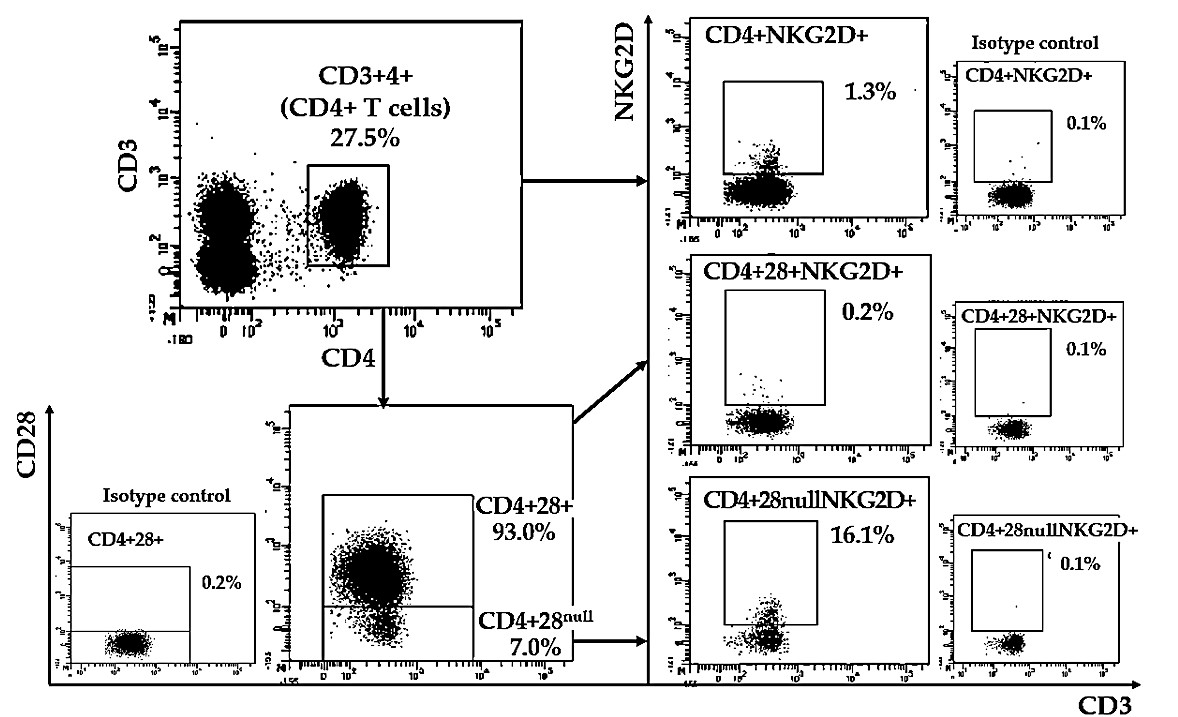

Supplement: Supplementary file 1 [file ijerph-18-03210-s001.zip › Supplementary Figure 1_2.jpg]

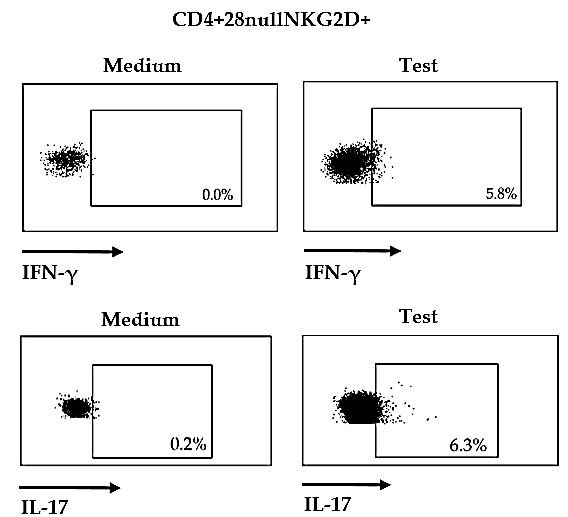

Supplement: Supplementary file 1 [file ijerph-18-03210-s001.zip › Supplementary Figure 2_2.jpg]
